# Primary Bone Lymphoma of the Jaw Masquerading as Infection and Delaying Treatment

**DOI:** 10.3390/hematolrep18010011

**Published:** 2026-01-22

**Authors:** Emily Hamburger, Anne W. Beaven

**Affiliations:** Lineberger Comprehensive Cancer Center, University of North Carolina, 101 Manning Drive, Chapel Hill, NC 27514, USA

**Keywords:** primary bone lymphoma, diffuse large B cell lymphoma, jaw

## Abstract

**Background:** Diffuse large B cell lymphoma is an aggressive, heterogeneous yet treatable disease. Primary bone lymphoma is a lymphoma involving a single or multiple osseous sites with or without regional nodal involvement. It is exceedingly rare, representing <1% of new non-Hodgkin lymphoma cases per year. Most cases of primary bone lymphoma are diffuse large B cell lymphoma. They infrequently involve the craniofacial bones and mandible; its rarity can lead to delays in diagnosis. **Case Series Presentation:** Two 64-year-old male patients initially presented to local dentists with concerns of tooth pain and numbness. Both underwent extensive dental procedures including extraction and debridement, with an initial diagnosis of osteomyelitis. They were placed on long-term antibiotics. After months without improvement, further testing was pursued, including imaging and repeat biopsies. The patients were finally diagnosed with primary bone diffuse large B cell lymphoma. From the initial treatment of osteomyelitis, a median time of 8.5 months passed before diagnosis of lymphoma. Treatment with cytotoxic chemotherapy was initiated and both patients achieved remission. **Conclusions:** As in the two cases presented here, the initial point of entry into the medical system may be a visit to the local dentist. When patients present with periodontal complaints, it is imperative to maintain a broad differential, including lymphoma. This is especially crucial when the patient’s clinical course does not respond to initial treatment. This results in delays of diagnosis and initiation of therapy for a treatable cancer.

## 1. Background

Diffuse large B cell lymphoma (DLBCL) is an aggressive and heterogenous disease. DLBCL represents 24% of new non-Hodgkin lymphoma cases per year. DLBCL typically presents as rapidly growing disease and may have concurrent B symptoms of fevers, night sweats, and significant weight loss. The majority of patients have disease only involving lymph node sites, but about a third of patients present with disease limited to extra-nodal sites such as the gastrointestinal tract, the thyroid, liver, kidney, or as in the cases presented here, to the cortical bone [1,2]. Primary bone lymphoma is a rare lymphoma involving a single or multiple osseous sites with or without regional nodal involvement. It is uncommon, representing <1% of new non-Hodgkin lymphoma (NHL) cases per year. Most cases of primary bone lymphoma are diffuse large B cell lymphoma (PBL-DLBCL) for which treatment typically includes cytotoxic chemotherapy–immunotherapy with or without radiation therapy for localized disease [3].

PBL-DLBCL infrequently involves the craniofacial bones with mandible involvement representing only 0.6% of all extra-nodal NHL. Its rarity can lead to delays in diagnosis. We present here two cases of PBL-DLBCL in the mandible that took a median time of 8.5 months to diagnose after initial treatment as osteomyelitis.

## 2. Case Presentation

Patient 1

A 64-year-old male with no significant past medical history had two teeth extracted in August 2023 due to spontaneous loosening. The recovery course was complicated by slow wound healing which necessitated two additional debridements. No biopsies or cultures were collected from these procedures. A course of antibiotics including metronidazole and clindamycin was prescribed for presumed infection.

Five months later, without resolution of these issues, imaging revealed extensive lytic destruction of the interior/buccal anterior mandible with concern for either osteomyelitis or malignancy. The patient underwent surgery with oral maxillofacial surgeons on 29 January 2024, with the extraction of more teeth, biopsy, and debridement. Pathology results were consistent with chronic inflammation and osteomyelitis with infiltration of lymphocytes, histiocytes, plasma cells, and rare neutrophils without evidence of malignancy; no special staining studies to rule out malignancy were reported. Although final cultures only grew oropharyngeal flora and his inflammatory markers including C-reactive protein and erythrocyte sedimentation rate were within normal limits, he received antibiotics for presumptive periapical/limited osteomyelitis of the mandible. Despite receiving antibiotics for 6 weeks, there was no improvement. Repeat biopsy performed at the end of April 2024, now 8 months from the initial dental problems, showed extensive areas of necrosis. In better preserved areas, there was a diffuse proliferation of abnormal lymphoid cells. These abnormal lymphoid cells were intermediate to large in size with irregular nuclear contours. The tumor cells were negative for S100 and CK-AE1/AE3 but positive for CD20 and CD10, leading to a diagnosis of germinal center diffuse large B cell lymphoma (CD30+ in 50%, EBV negative, Ki67 50–60%, not double expressor or double hit).

**Figure 1:**
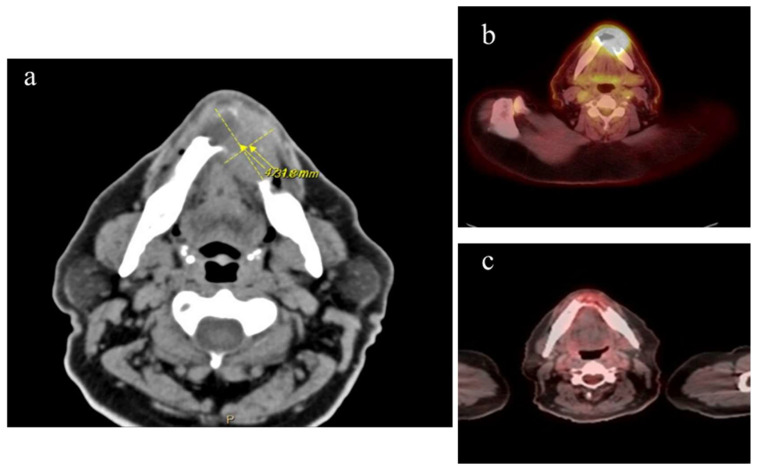
A 64-year-old M with DLBCL stage IE. (**a**) CT imaging of mandible with mass and associated bone destruction at the time the lymphoma diagnosis was made, 9 months after initial presentation with loose teeth and shortly after completing a 6-week course of oral antibiotics. (**b**) PET scan taken during initial lymphoma staging, with uptake in mandibular area SUV 18.8. (**c**) PET scan after completion of 3 cycles of chemotherapy, showing complete remission.

Staging confirmed that he had stage IE CD30+ DLBCL with soft tissue/mandible involvement and an IPI of 1 (Figure 1). Chemotherapy was initiated with rituximab, brentuximab vedotin, cyclophosphamide, vincristine, and prednisone. He was in complete remission after three cycles of chemotherapy followed by consolidative radiation treatment.

Patient 2

A 64-year-old male first noted dental pain in early 2020. He was admitted to the hospital in December 2020 due to worsening dental pain, face and chin numbness, and facial swelling. CT imaging showed a multiloculated subperiosteal abscess along the right mandible as well as multiple enhancing and necrotic cervical nodules. The patient was emergently brought to the operating room for incision and drainage of the right submandibular, sublingual, and vestibular spaces as well as teeth extraction. A bone biopsy was completed, and pathology was suggestive of osteomyelitis with bone destruction; no special staining for malignancy was reported. Cultures were negative. The patient was discharged from the hospital with a six-week course of IV Vancomycin and Unasyn. He self-discontinued the antibiotics after a week and subsequently agreed to oral antibiotics for treatment of the right mandibular abscess/osteomyelitis.

**Figure 2:**
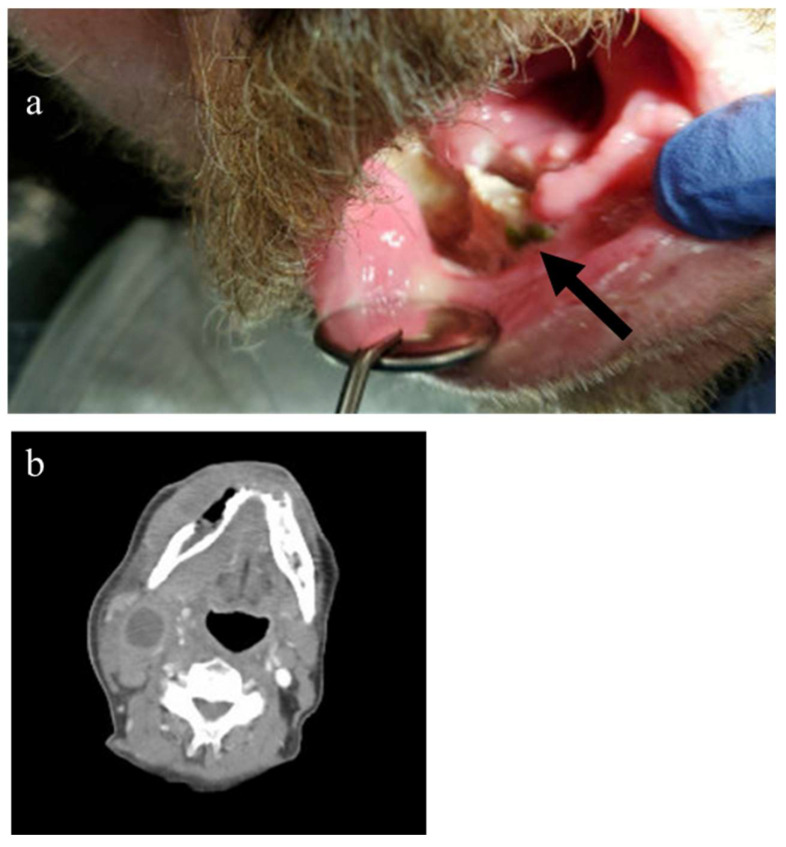
A 64-year-old man with DLBCL stage IIeb at the time the lymphoma diagnosis was made, 2 months after 1st biopsy and after no improvement with systemic antibiotics. (**a**) Photo of right lower oral cavity site of tumor (black arrow). (**b**) CT scan taken during staging, with mass and associated bone destruction in yellow circle and necrotic cervical nodes red arrow.

His symptoms did not improve on antibiotics, and he noted a progressive enlargement of a mass on his right neck. A repeat CT scan on 5 January 2021 was significant for an oral mass and cervical lymphadenopathy. Biopsy of contiguous floor of mouth lesions showed sheets of malignant cells with cellular pleomorphism and atypical mitoses. The cells were poorly differentiated, requiring extensive work up in which a negative AE1/3 and CAM5.2 keratin marker ruled out prostate cancer and negative SOX10 ruled out melanoma and peripheral nerve sheath tumor. A variable staining CD44 suggested a possible lymphoma with strongly positive CD10, CD20, and kappa light chain restriction, leading to diagnosis of germinal center diffuse large B cell lymphoma (myc negative). PET scan on 4 February 2021 showed intense radiotracer uptake (SUV 7.80) diffusely along the right mandibular body and ramus, muscles of the right lower jaw, and conglomeration of right cervical lymph nodes consistent with stage IIE disease (Figure 2). He underwent rituximab, cyclophosphamide, doxorubicin, vincristine, prednisone for five cycles and completed the regimen on June 2021. The sixth cycle was not pursued due to multiple hospitalizations for pulmonary issues after cycle five. The PET scan after completing chemotherapy demonstrated that he was in a complete remission. He remained disease free for three years when he died from unrelated causes.

## 3. Discussion

PBL–DLBCL subtypes are rare extra-nodal lymphomas These account for <1% of all lymphomas and about 7% of all bone malignancies. At diagnosis, half of all patients have Stage I disease, limited to one skeletal site. The most frequent areas of involvement are the axial and appendicular skeleton, with craniofacial involvement accounting for only 8.8% of bone lymphoma cases [4]. PBL-DLBCL can occur at any age, but it most commonly occurs between ages 40–60 [2].

Pain is the most frequent initial symptom with a median duration of symptoms prior to diagnosis of over 3 months [5]. Patients can also present with masses, neurologic symptoms, or pathological fractures [6]. Interestingly, systemic symptoms such as fever, night sweats, and weight loss are not commonly present at diagnosis [3]. It is typical that PBL DLBCL arising in the oral cavity is accompanied by symptoms mimicking infection such as bony destruction. Other symptoms such as loss of sensation, tooth instability, and ulceration are also common with initial presentation [7].

Due to the non-specific symptomology of PB-DLBCL in the mandible as well as its rarity, dentists and physicians have a low index of suspicion for this diagnosis. As seen with these cases, patients with PBL-DLBCL often undergo unnecessary endodontic procedures and long-term antibiotics before the proper diagnosis is identified [7]. Overall response rate for treatment of PBL-DLBCL is 91%, with a complete response rate of 74% in patients with Stage I and II PBL [5]. Patients with an early stage of the disease have excellent prognosis, emphasizing the necessity of early diagnosis.

Despite significant symptoms, both patients’ courses were complicated by misdiagnosis of osteomyelitis and unnecessary use of long-term antibiotics. Final diagnosis took an average of 8.5 months, therefore delaying appropriate care in the setting of an aggressive cancer. This is consistent with previous reports of 27 cases of extra-nodal DLBCL involving the maxilla or mandible in which there was a median of 60 days from development of symptoms until pursuing treatment. A total of 2 of the 27 patients were initially diagnosed with osteomyelitis and, like our patients, had significant delays (90 and 360 days) from onset until treatment. Also, about 60% of patients underwent dental procedures before referral for further work up [7].

The current standard of treatment involves chemotherapy with rituximab, cyclophosphamide, doxorubicin, vincristine, and prednisone (R-CHOP) or similar regimens plus or minus radiation. Surgery is not part of the treatment for DLBCL but is sometimes required when pathological fractures are present [3]. One case series reported a complete response rate with RCHOP +/− radiation of 80%, a 4-year overall survival of 83%, and progression free survival of 74.5% [8].

Both patients in our case series received a regimen based on R-CHOP. One patient received consolidative radiation and one patient received brentuximab vedotin due to the tumor being CD30+. Treatment led to complete remission for both patients.

## 4. Conclusions

As in the cases presented here, the initial point of entry into the medical system is often a visit to the dentist or oral surgeon. When patients present with periodontal complaints, it is imperative to maintain a broad differential, especially if there is no response to the initial treatment. This results in delays of diagnosis and initiation of therapy for a treatable cancer.

## Data Availability

The original contributions presented in this study are included in the article. Further inquiries can be directed to the corresponding author.

## Abbreviations

DLBCL	Diffuse large B cell lymphoma
NHL	Non-Hodgkin lymphoma
PBL-DLBCL	Primary bone lymphoma—diffuse large B cel lymphoma
R-CHOP	Rituximab, cyclophosphamide, doxorubicin, vincristine, prednisone
